# Profile of blood cell abnormalities among antiretroviral therapy naïve HIV patients attending the Yaounde University Teaching Hospital, Cameroon

**DOI:** 10.1186/2052-1839-14-15

**Published:** 2014-09-08

**Authors:** Paul Nji Wankah, Claude Tayou Tagny, Dora Ngum Shu Mbanya

**Affiliations:** Faculty of Medicine and Biomedical Sciences Yaoundé, University of Yaoundé 1, P.O Box 1364, Yaoundé, Cameroon; Hematology Laboratory of the Yaounde University Teaching Hospital and Faculty of Medicine and Biomedical Sciences, University of Yaoundé 1, Yaounde, Cameroon

**Keywords:** HIV, Antiretroviral therapy naive, Peripheral blood cell abnormalities, AIDS, Hemogram, Reticulocyte count

## Abstract

**Background:**

Abnormal hemograms are common manifestations and important predictive tools for morbidity in the human immunodeficiency virus (HIV) infection. Few studies have been reported on the blood profile of HIV antiretroviral therapy (ART) naive subjects, therefore this study aimed to quantitatively and qualitatively describe the blood cell profile of HIV ART naive patients, and to describe the occurrence of the blood cytopenias by CD4 cell counts and WHO clinical stage.

**Methods:**

This cross-sectional study of ART naive HIV patients was done at the Yaounde University Teaching Hospital (YUTH). For eligible participants, a structured questionnaire was filled and a clinical examination was done. Blood samples were collected for the measurement of full blood count and CD4 cell count. Blood films were made for the cytological examination of the blood samples and a reticulocyte count was done by the cresyl blue stain method.

**Results:**

Of 81 cases reviewed, 66 (81.5%) had a blood cell disorder. The main qualitative blood disorders on the blood film were metamyelocytes (37.1%), toxic neutrophils (33.3%), stab neutrophils (29.6%), anisocytosis (35.6%) hypochromia (32.1%) and giant platelets (22.2%). Anaemia (62.9%) was the most common quantitative disorder of which 86.3% had low reticulocyte counts. Participants with low CD4 counts and advanced clinical stages had a greater occurrence of blood cytopenias (p-values <0.05).

**Conclusion:**

In the HIV infection, peripheral blood cell abnormalities affect all cell lineages, with anaemia being the most frequent single blood cell abnormality. Blood cytopenias mainly occur in advanced immunosuppression and clinical stages. Although all HIV patients may have blood cell disorders, those with advanced disease are more prone to develop them.

## Background

The Human Immunodeficiency Virus (HIV) infection causes the Acquired Immunodeficiency Syndrome (AIDS), which is a systemic disorder characterized by severe deficiency in cellular immune responses. Besides infectious complications, several peripheral blood cell abnormalities have been reported in HIV infection, of which anaemia and neutropenia are reportedly the most common [1–4].

Amid the multifactorial causes of anaemia and neutropenia, several studies agree that inadequate hematological production due to bone marrow suppression by HIV infection through abnormal cytokine expression, and the alteration of the bone marrow microenvironment is preponderant [5–7]. On the other hand, thrombocytopenia is usually caused by immune-mediated destruction of platelets, in addition to inadequate production [8]. Other causes of cytopenias in HIV infected patients include treatment-related adverse events, opportunistic infections, malignancies, drugs (zidovudine and cotrimoxazole) and other pre-existing or co-existing medical problems [3, 9, 10]. Anaemia in HIV-infected persons is associated with CD4 cell depletion and progression to AIDS and is one of the strongest predictors of poor responses to antiretroviral therapy (ART) and HIV-related mortality [1, 11].

Hematological abnormalities have been documented as strong independent predictors of morbidity and mortality in HIV infected individuals [1, 3, 12]. Although it is not part of the criteria for initiating therapy nor used by the World Health Organization (WHO) for staging HIV, peripheral blood cell abnormalities in an abnormal hemogram are important prognostic tools for morbidity in HIV infection and AIDS [1, 3]. There are several reports that anaemic HIV patients have a higher morbidity and mortality rate than those without anaemia [13–15].

Few studies have been done in sub-Saharan Africa on the peripheral blood cell abnormalities of HIV infected persons, despite them being common manifestations of HIV infection and AIDS, which may have a considerable impact on the patient’s wellbeing, and treatment (antiretroviral regimens containing zidovudine would not be used by patients having severe anaemia or pancytopenia). The 2001 Mbanya et al. [16] study at the Central Hospital of Yaounde, done among ART naive AIDS patients revealed that 95.4% of AIDS patients had morphological blood cell disorders compared to 32.2% amongst healthy controls. Thus, given that ART naive AIDS patients have a significantly high prevalence of peripheral blood abnormalities, this study was carried out at the Yaounde University Teaching Hospital (YUTH) of Cameroon to further determine if this was alike for HIV ART naive patients of all WHO clinical stages and immunological classes (CD4 counts), and also to determine if the clinical or immunological severity of HIV would predispose to peripheral blood cytopenias.

## Methods

### Study setting and study population

This was a cross-sectional study of antiretroviral therapy naive HIV-infected people who enrolled at the HIV clinic of the Yaounde University Teaching Hospital (YUTH) between August 2013 and February 2014. Consenting HIV infected adults (>18 years) who were not yet on antiretroviral medications, were recruited consecutively into the study. Pregnant HIV infected adults, and those on tuberculosis medication or cotrimoxazole prophylaxis were excluded from the study.

### Ethical considerations

Ethical clearance was obtained from the National Ethical Committee, Cameroon. Permission for the conduct of the study was obtained from the Yaounde University Teaching Hospital. After informing study participants of the objectives of the study and assuring them of confidentiality of their data, written informed consent was taken from all the participants.

### Data collection

Data on the socio-demographic characteristics of the participants was collected using a pre-tested and structured questionnaire by interview. Clinical data was collected during a clinical examination, when symptoms and signs that define the WHO clinical stage of HIV disease [17] were recorded on the questionnaire.

For the full blood count (FBC), blood smear and reticulocyte count, blood was collected into a tube containing Ethylene Diamine Tetra- Acetate (EDTA) at the YUTH hematology laboratory. The FBC were performed using the HumaCount Plus automated blood analyser (Human GmbH, Weisbaden, Germany) less than 2 hours after venesection. Reticulocyte count was done by standard methods [18] and the blood smear was stained by the MayGrunwald and Giemsa method [19] and read by a pathologist.

The participants were then referred to the Chantal Biya International Reference Center (CIRCB) where the CD4 count was done using the Becton Dickinson FACSCalibur system (Becton Dickinson, Singapore).

Administrative authorisations were obtained from the directors of both centers for the usage of their facilities.

### Statistical analysis

The occurrence of qualitative peripheral blood cell abnormalities were expressed as proportions or percentages and were classified as categorical variables.

Reticulocyte counts were used to qualify anaemia as regenerative or aregenerative (aregenerative anaemia with reticulocyte counts <150,000/mm^3^) [18].

The Chi-square test was used to assess the presence of statistically significant association between the quantitative peripheral blood cell abnormalities, anaemia (Hemoglobin < 12 g/dl for women and < 13 g/dl for men) [20], leucopenia (leucocyte count <4 × 10^9^ cells/L) [21] and thrombocytopenia (platelet count < 150 × 10^9^ cells/L) [21], and their occurrence in the different WHO HIV clinical stages [17] and WHO HIV immunological classes [17]. In all cases P-value less than 0.05 was considered as statistically significant.

Statistical analyses were performed using the Epi Info 7 statistical software.

## Results

### General characteristics of study participants

During the study period we identified 124 ART naïve HIV infected people, and 43 were excluded because they were either on tuberculosis treatment, cotrimoxazole prophylaxis or pregnant. The remaining 81 cases were reviewed consisting of 53 (65.4%) women and 28 (34.6%) men. The mean age of the participants was 37.9 ± 10.8 years, range 22–70. All the participants had never been on antiretroviral therapy (Table 1).

**Table 1:** **Characteristics of antiretroviral naïve HIV positive adult patients at the Yaounde University Teaching Hospital Clinic from August 2013-February 2014**

Variables	HIV patients (n = 81)
**Age**	
< 25 years	15 (18.6%)
25-45 years	49 (60.5%)
> 45 years	17 (20.9%)
**Sex**	
Male	28 (34.6%)
Female	53 (65.4%)
**Marital status**	
Married	22 (27.1%)
Single	39 (48.2%)
Divorced	8 (9.9%)
Widow(er)	12 (14.8%)
**WHO Clinical stage**	
I	38 (46.9%)
II	19 (23.5%)
III	11 (13.5%)
IV	13 (16.1%)
**WHO Immunological class (CD4 cell count)**	
Not significant immunosuppression (>500/mm3)	16 (19.7%)
Mild immunosuppression (350-499/mm3)	19 (23.5%)
Advanced immunosuppression (200-349/mm3)	20 (24.6%)
Severe immunosuppression (<200/mm3)	26 (32.2%)

The majority of our research participants, 38 (46.9%), were asymptomatic, while 26 (32.2%) had severe immunosuppression (Table 1).

### Peripheral blood cell parameters and abnormalities

66 of the participants had at least one quantitative or qualitative peripheral blood cell disorder, giving a prevalence of 81.5%.

Table 2 shows that the mean leucocyte and platelet counts were within the normal range, while the mean haemoglobin concentration (10.8 ± 2.1 g/dL) was lower than normal. The mean CD4+ cell count was 298.7 ± 216.2 cells/mm^3^.

**Table 2:** **Full blood count results of antiretroviral naïve HIV positive adult patients at the Yaounde University Teaching Hospital Clinic from August 2013-February 2014**

Parameter (n = 81)	Mean value ± SD	Range (min-max)
**Total WBC x 10** ^**9**^ **/L**	5.1 ± 1.8	1.6-10.6
Neutrophils	2.4 ± 1.2	0.7-5.1
Monocytes	0.4 ± 0.3	0.1-1.1
Lymphocytes	1.9 ± 1.3	0.3-6.7
**Total RBC x10** ^**12**^ **/L**	4.4 ± 0.9	2.2-6.6
Haemoglobin (g/dl)	10.8 ± 2.5	5.1-15.8
Hematocrit (%)	33.6 ± 7.3	17.1-50.0
MCV (fl)	77.1 ± 10.1	53.0-95
MCH (pg)	25.2 ± 4.2	16.7-33.9
MCHC (g/dl)	32.0 ± 1.8	29.6-36.0
**Reticulocyte x 10** ^**9**^ **/L**	44.5 ± 34.1	8-153
**Total platelet count x10** ^**9**^ **/L**	256.2 ± 113.9	35.0-556.0

*WBC* white blood cell count, *RBC* red blood cell count, *MCV* mean corpuscular volume, *MCH* mean corpuscular haemoglobin, *MCHC* mean corpuscular haemoglobin concentration.

Anaemia was the most frequent single quantitative hematological abnormality, occurring in 51 (62.9%) cases, of which 44 (86.3%) were aregenerative. Leucopenia occurred in 28 (34.6%) cases while thrombocytopenia occurred in 22 (27.1%) cases.

Qualitative disorders occurred in all the peripheral blood cell lineages, with the most frequent being the presence of metamyelocytes, toxic neutrophils, anisocytosis, hypochromia and giant platelets on the blood films (Table 3).

**Table 3:** **Frequency of occurrence of qualitative blood cell disorders on blood film per lineage**

Type of variation	Number of cases (n)	Percentage (%)	95% CI
***Leucocyte lineage***			
**Metamyelocytes**	30	37.1	26.6-48.5
**Toxic neutrophils**	27	33.3	23.2-44.7
**Stab neutrophil**	24	29.6	19.9-40.8
**Pelger-like neutrophil**	24	29.6	19.9-40.8
**Myelocyte**	13	16.1	8.8-25.8
**Hypogranular neutrophils**	12	14.8	7.9-24.4
**Hypersegmented neutrophil**	10	12.4	6.1-21.5
**Atypical lymphocytes**	10	12.4	6.1-21.5
**Binucleated lymphocyte**	2	2.5	0.3-8.6
***Erythrocyte lineage***			
**Anisocytosis**	28	35.6	24.3-45.9
**Hypochromia**	26	32.1	22.1-43.4
**Microcytosis**	22	27.2	17.9-38.2
**Poikilocytosis**	10	12.6	6.1-21.5
**Nucleated red cells**	4	4.94	1.4-12.2
**Macrocytes**	2	2.5	0.3-8.6
**Red cell inclusion**	1	1.3	0.03-6.7
**Polychromasia**	1	1.3	0.03-6.7
***Thrombocyte lineage***			
**Giant platelets**	18	22.2	13.7-32.8
**Clustered platelets**	8	9.9	4.4-18.5

*N* total cases, *n* number of participants having the cytological abnormality on the blood film.

### The occurrence of the peripheral blood cytopenias in the WHO clinical and immunological stages

Tables 4 and 5 both show that anaemia, leukopenia and thrombocytopenia are all strongly and directly related to CD4 cell counts and WHO disease stage, where the lowest prevalence of blood cytopenias is among those with clinical stage 1/CD4 cell count >500/μL, and the highest prevalence of all three cytopenias is among those with severe immunosuppression and stage 4 disease (p < 0.05 for all). With the exception of leucopenias by WHO disease stage, these all demonstrate graded associations.

**Table 4:** **The occurrence of peripheral cytopenias in the different WHO immunological classes**

WHO Immunological class	Anaemia	Leucopenia	Thrombocytopenia
(CD4 cells/μl) [N]	(n) %	(n) %	(n) %
**No deficiency (>500) [16]**	(7) 43.8	(3) 18.8	(0) 0
**Mild (350–499) [19]**	(9) 45.0	(5) 25.0	(4) 20
**Advanced (200–350) [20]**	(13) 68.4	(6) 31.6	(4) 21
**Severe (<200) [26]**	(22) 84.6	(14) 58.9	(14) 53.8
**P-values**	<0.05	<0.05	<0.05

*WHO* World Health Organization, *N* number of participants per immunological class, *n* number of participants per immunological class with a given cytopenia.

**Table 5:** **The occurrence of peripheral cytopenias in the different WHO clinical stages**

WHO Clinical stage [N]	Anaemia	Leucopenia	Thrombocytopenia
	(n) %	(n) %	(n) %
**Stage 1 [38]**	(19) 50.0	(9) 23.7	(2) 5.3
**Stage 2 [19]**	(11) 57.9	(5) 26.3	(2) 10.5
**Stage 3 [11]**	(8) 72.7	(7) 63.6	(8) 72.7
**Stage 4 [13]**	(13) 100	(7) 53.8	(10) 76.9
**P-values**	<0.05	<0.05	<0.05

*WHO* World Health Organization, *N* number of participants per immunological class, *n* number of participants per immunological class with a given cytopenia.

## Discussion

Peripheral blood cell abnormalities in HIV ART naive patients are multifactorial in nature (immune mechanisms, drugs, opportunistic infections or direct insult of HIV). The 81.5% prevalence of qualitative and quantitative peripheral blood cell disorder in the hemogram of ART naïve HIV patients in this study concurs with the findings of a similar study on AIDS patients [16]. Mbanya et al. [16] worked on symptomatic HIV patients who probably had severe immune deficits, meanwhile 46.9% of our research participants were asymptomatic, reinforcing the observation that HIV causes peripheral blood cell disorders in all infected patients indiscriminate of clinical stage or CD4 cell count.

The prevalence of anaemia, leucopenia and thrombopenia in this study variably concurs and contrasts with the findings of similar studies [4, 22, 23]. Possible reasons for the differences of occurrence of these cytopenias in these different studies are the cut-off values used for anaemia and leucopenia, the local prevalence of parasitic infections such as malaria or hook-worms, variations in local nutritional patterns or the number of female participants of the respective studies. There is also a racial disparity, with Africans having lower leucocyte counts.

Although all HIV infected patients may have a peripheral blood cytopenia, patients with advanced disease and low CD4 counts (Tables 4 and 5) are more likely to develop them, as reported in several studies [8, 24–26]. This is important for caregivers at the HIV clinic, because some drugs like zidovudine and cotrimoxazole are myelotoxic and should not be administered to patients with severe cytopenias, thus all HIV patients, especially those with advanced disease, should systematically do a full blood count before initiation of antiretroviral therapy.

Given that anaemia in ART naive HIV patients is mostly aregenerative with reticulocyte counts less than 150 × 10^9^/L [3], HIV caregivers should orientate etiological investigations towards causes of inadequate bone marrow response which may be, bone marrow suppression due to the HIV virus (upregulation of cytokines in the bone marrow), nutrient deficiency, chronic opportunistic infections (tuberculosis, *Mycobacterium avium complex*, cytomegalovirus) or drugs (acyclovir, cotrimoxazole). Appropriate management of anaemia in HIV depends on a good etiological diagnosis which starts by knowing if it is peripheral or central origin.

The erythrocyte lineage abnormalities observed on the blood films (Table 3) may result from iron deficiency anaemia which could be due to parasitic infections or nutritional deficiency [3]. Being in a tropical region, parasitic infections [27] could contribute to the occurrence of this anaemia. Macrocytosis which is often associated with zidovudine and stavudine treatment regimens was not a feature of these patients since our study group were naïve for antiretroviral treatment. Furthermore folate deficiency, vitamin B 12 deficiency, dyserythropoiesis and megaloblastic maturation which has been reported by several investigators [3, 16, 24, 25] as a cause of anaemia in HIV patients could explain the 2.5% frequency of macrocytosis in the study. The morphological abnormalities found amongst the leucocytes were mainly of the granulocytic lineage with the presence of discrete numbers of young cells, but also the presence of toxic neutrophils (33.3%) and pelger-like neutrophils (29.6%) which indicate bacterial infections. These findings are similar to those of Mbanya et al. [16] who had a prevalence of 34% for pelger-like neutrophils amongst the AIDS group, and which was significantly different from the control group. The major platelet abnormalities were giant platelets which represented 22.2% in this study. Given that there are qualitative peripheral blood cell disorders affecting all the cell lineages in HIV infection, and there is no pathognomonic morphological blood cell abnormality in HIV infection, it is important that pathologists include HIV as differential diagnoses of abnormal blood films (especially in high prevalence areas).

Because of a lack of resources, this study was limited in that the sample size was small and it does not address hemoglobinopathies. Also, there was no comparison done to an HIV-uninfected group in Yaounde to show what blood cell disorders may be driven by local factors versus HIV-infection. Despite this, the study provides valuable information on the peripheral blood cell profile of antiretroviral naïve HIV patients focusing on the quantitative and qualitative disorders as read on the blood film by a pathologist. Reticulocyte counts which are not systematically done in our setting were included in this study, so as to determine if anaemia was of central or peripheral origin. It would be interesting to study other hematological parameters (like coagulation profile) that may be altered in antiretroviral naïve HIV patients.

## Conclusion

Several quantitative and qualitative peripheral blood cell disorders occur in ART naïve HIV infected patients, the most common of which is anaemia. These peripheral blood cell disorders occur in all WHO clinical and immunological HIV stages and they are most common in people with profound and advanced immune deficiencies. It is important that blood pathologist include HIV as a differential diagnosis of an abnormal hemogram, given the high prevalence cytological disorders in the hemogram of HIV infected individuals.

We thus recommend at the end of this study that physicians should screen for hematological abnormalities in all HIV infected patients, especially those with advanced disease, and health regulators should provide automatic blood counters that systematically do reticulocyte counts in HIV clinics.

## Abbreviations

AIDS: Acquired immunodeficiency syndrome
ART: Antiretroviral therapy
BD FACS: Becton dickinson fluorescence activated cell sorter
CD4: Cluster of differentiation 4
CIRCB: Chantal biya International reference center
EDTA: Ethylene diamine tetra- acetate
FBC: Full blood count
HIV: Human immunodeficiency virus
MCV: Mean cell volume
MCHC: Mean corpuscular hemoglobin concentration
MCH: Mean corpuscular hemoglobin
HCT: Hematocrit
RBC: Red blood cell
WBC: White blood cell
WHO: World Health Organisation
YUTH: Yaounde jniversity teaching hospital.
